# Single-Cell Analysis and Next-Generation Immuno-Sequencing Show That Multiple Clones Persist in Patients with Chronic Lymphocytic Leukemia

**DOI:** 10.1371/journal.pone.0137232

**Published:** 2015-09-09

**Authors:** Jitra Kriangkum, Sarah N. Motz, Tanner Mack, Sara Beiggi, Eva Baigorri, Hemalatha Kuppusamy, Andrew R. Belch, James B. Johnston, Linda M. Pilarski

**Affiliations:** 1 Department of Oncology, University of Alberta and Cross Cancer Institute, Edmonton, Canada; 2 Manitoba Institute of Cell Biology, Winnipeg, Canada; Jackson Laboratory, UNITED STATES

## Abstract

The immunoglobulin heavy chain (*IGH)* gene rearrangement in chronic lymphocytic leukemia (CLL) provides a unique molecular signature; however, we demonstrate that 26/198 CLL patients (13%) had more than one *IGH* rearrangement, indicating the power of molecular technology over phenotypic analysis. Single-cell PCR analysis and next-generation immuno-sequencing identified *IGH*-defined clones. In 23% (18/79) of cases whose clones carried unmutated immunoglobulin heavy chain variable (*IGHV*) genes (U-CLL), *IGH* rearrangements were bialleic with one productive (P) and one non-productive (NP) allele. Two U-CLL were biclonal, each clone being monoallelic (P). In 119 *IGHV*-mutated (M-CLL) cases, one had biallelic rearrangements in their CLL (P/NP) and five had 2–4 distinct clones. Allelic exclusion was maintained in all B-clones analyzed. Based on single-cell PCR analysis, 5/11 partner clones (45%) reached levels of >5x10^9^ cells/L, suggesting second CLL clones. Partner clones persisted over years. Conventional *IGH* characterization and next-generation sequencing of 13 CLL, 3 multiple myeloma, 2 Waldenstrom’s macroglobulinemia and 3 age-matched healthy donors consistently identified the same rearranged *IGH* sequences. Most multiple clones occurred in M-CLL, perhaps indicative of weak clonal dominance, thereby associating with a good prognosis. In contrast, biallelic CLL occurred primarily in U-CLL thus being associated with poor prognosis. Extending beyond intra-clonal diversity, molecular analysis of clonal evolution and apparent subclones in CLL may also reflect *inter*-clonal diversity.

## Introduction

Chronic lymphocytic leukemia (CLL) is characterized by a monoclonal B-cells having a unique immunoglobulin heavy chain (*IGH)* gene rearrangement. Mutational status of the clonotypic immunoglobulin heavy variable (*IGHV*) gene stratifies CLL patients into two groups. In about 60% of cases the *IGHV* is mutated (M-CLL) while 40% are in germline configuration (U-CLL). In general, patients with U-CLL have a worse prognosis than those with M-CLL. The cellular origin(s) of CLL clone remains unresolved but recent DNA methylation studies have suggested that the U-CLL cell is more similar to a naïve B-cell, with M-CLL being similar to a memory B-cell [1].

Multiple productive *IGH* rearrangements (P) have been reported in a subset of CLL [2]. It is unclear whether these are derived from distinct/unrelated clones or if two productive rearrangements arise in a single B-CLL cell. The rule of allelic exclusion demands that each cell harbors only one productive rearrangement. If the first attempt at *IGH* rearrangement fails, the second allele is then allowed to rearrange; if the second allele fails to yield a productive rearrangement, the B-cell dies. A previous study suggested that CLL cells may not follow this rule and the presence of two productive *IGH* rearrangements in a single cell could result from *IGHV* gene replacement [3, 4]. A more recent study however suggested that multiple productive *IGH* rearrangements in CLL may represent multiple independent clones, as suggested by light chain restriction or phenotype [5]. In support of this latter hypothesis are the observations that, by immunophenotyping, biclonal CLL is seen in a small percentage of patients [5–11]. In addition, unique molecular and cytogenetic features characterized phenotypically distinct clones coexisting in MBL, CLL and other B-cell lymphoproliferative disorders [12, 13]. In spite of these collective data, the absence of single-cell analysis (SCA) in most studies has made it difficult to pinpoint the distinct clones especially those minor but still frequent clones that are likely to be missed by phenotyping, or clones that cannot be distinguished phenotypically.

Aberrant and recurrent mutations have been reported in multiple genes using conventional Sanger sequencing as well as genome-wide next-generation sequencing, suggesting that certain recurrent mutated genes contribute to clonal evolution and disease progression in CLL [14–16]. Given that even very small sub-clones appear to have a significant negative impact on outcome [17], this may be clinically important. And while it is believed that these subclones are related to the primary CLL clone, recent studies suggest that they may reflect small secondary clones which have a survival and growth advantage over the primary clone [5].

In the present study, we molecularly determined the incidence of multiple productive rearrangements in CLL, their clonal origin and their persistence throughout the course of disease. CLL patients identified as harboring more than one *IGH* rearrangement were analyzed to determine whether this represented bialleic rearrangements in the same host cell or distinct B-cell clones (bi- or multiclonality). Partner clones were confirmed using next-generation *IGH* sequencing (NGS) and their frequencies among B-cells were verified using SCA. For this cohort of patients, we found that the rules of allelic exclusion were maintained in all clones analyzed. Partner clones arose in both U-CLL and M-CLL, with a trend towards multiple clones among patients with M-CLL. In contrast, monoclonal disease with biallelic *IGH* typically arose in U-CLL. For patients with multiple independent clones, the partner clones were detected among very large numbers of the “primary” CLL clone, indicating that their frequencies exceed that of any normal B-cell population. Some partner clones exceeded 5x10^9^ cells/L and were persistent over time and with treatment. Thus, in addition to potential *intra*-clonal diversity, molecular analysis of clonal evolution and apparent subclones in CLL may also reflect *inter*-clonal diversity.

## Patients, Materials and Methods

### Patients

CLL was diagnosed based on consensus criteria, with typical CLL-type monoclonal B-cells (CD19^+^CD5^+^CD23^+^) [18]. Anonymous samples were from the Manitoba Institute of Cell Biology Tumor Archive. Three age-matched healthy donors (HD) were anonymous. Three multiple myeloma (MM) [19] and two Waldenstrom’s macroglobulinemia (WM) [20] were from the Cross Cancer Institute. The study was approved by Health Research Ethics Board of Alberta and University of Manitoba Research Ethics Boards, after written informed consent in accordance with the Declaration of Helsinki. Clinical characteristics of the 198 randomly selected CLL patients are summarized in Table 1. The cutoff for designating U-CLL or M-CLL was the 2% mutation frequency in *IGHV* genes.

**Table 1:** Summary of CLL patient characteristics ^a^.

Number of patients	198
Male:Female ratio	1.7:1
Age, yr: median (range)	69 (37–91)
*IGHV* mutational status	
Unmutated	79 (40%)
Mutated	119 (60%)
Monoclonal B-cell lymphocytosis (MBL; typical CLL type)	1
Small lymphocytic lymphoma (SLL)	4
CLL Patients	193
Stage 0	97 (50%)
Stages I/II	74 (38%)
Stages III/IV	18 (9%)
Unknown	4 (2%)

^a^ The definitions of CLL, SLL and MBL are from the international workshop on CLL [18].

### Samples

Peripheral blood CLL lymphocytes were stored as a frozen cell pellet and aliquots were cryopreserved. Samples with a high lymphocyte count (>40x10^9^ cells/L) were not fractionated. Those with low counts (10-40x10^9^ cells/L) were B-cell enriched by negative selection using the RosetteSep Human B-Cell Enrichment Cocktail (STEMCELL Technologies, Vancouver, BC, Canada). Those with lymphocyte counts <10x10^9^ cells/L had positive CD19 selection.

### Complementary determining region 3 (CDR3) analysis

CDR3 analysis, primer sequences and calculation of CDR3 length followed Kriangkum *et al* [20]. CDR3 regions were amplified from gDNA using a fluorescence labeled FR3/JHc primer set. DNA fragment analysis was run on an ABI Prism 3130*xl* Genetic Analyzer (Applied Biosystems, Burlington, ON, Canada) and data was analyzed by GeneMapper software v4.0. PCR products were also cloned using TOPO TA cloning kit (Invitrogen) and sequence analysis was performed using BigDye Terminator v3.1 reagent (ABI) following the manufacturer instructions.

### Identification of clonotypic *IGH* sequences

This procedure followed Taylor *et al* [21]. All clonotypic *IGH* sequences were amplified from gDNA using primer sets that bind to leader sequences of *IGHV* gene families and *IGHJ* region. The most prominent rearranged *IGH* products were sequenced and confirmed by matching with the CDR3 analysis. Mutational and junctional analysis was performed using IMGT/V-QUEST program version 3.2.30 [22]. The primers for each rearranged *IGH* were designed based on unique CDR1 (sense) or CDR2 (sense) and CDR3 (antisense) sequences. Primers were tested specific against the clone of interest and without cross reactivity to various other B-clones of the same *IGHV* gene family. The selected primer set was used for clonal identification in SCA.

### Cell sorting and SCA

Cryopreserved samples were thawed, maintained overnight in culture medium at 37°C, 5%CO_2_. CD19^+^ cells were sorted into PCR tubes at a frequency of 1, 10 or 100 cells/tube using an Influxcell sorter (BD Biosciences, Mississauga, ON, Canada). Sorted single cells were analyzed by nested PCR [19]. Analysis was performed in 16–24 individual cells in samples that are monoclonal biallelic. For those samples with multiple clones, SCA was carried out in 96–110 individual cells. Clonal frequency was calculated as the percentage of cells positive in the test reaction over the total number of cells positive for β2 microglobulin (β_2_m). Analysis of 10 and 100 cell-aliquots was performed to validate those with low clonal frequencies. The frequency was interpreted as the presence of at least one clonal cell in the aggregate pool of cells analyzed (e.g., if 10 tubes with 100 cells each—a total of 1000 cells—are analyzed and only one tube shows positivity, the frequency is estimated to be at least 1/1000).

### Repertoire analysis by NGS

*IGH* CDR3 regions were amplified and sequenced by Adaptive Biotechnologies Corp (Seattle, WA, USA) using ImmunoSEQ, a multiplex PCR system used to amplify CDR3 sequences from gDNA samples [23–25]. Amplicons were sequenced using the Illumina HiSeq platform. Raw sequence data was filtered based on the *IGHV*, *IGHD* and *IGHJ* gene definitions provided by the IMGT database (www.imgt.org) and binned using a modified nearest-neighbor algorithm to merging closely related sequences and remove both PCR and sequencing errors. Data was analyzed using the ImmunoSEQ analyzer toolset.

### Statistical analysis

Data management and analysis was performed using Microsoft Excel (Microsoft Corp., Redmond, WA, USA) and SAS 9.2 (SAS Institute Inc., Cary, NC, USA).

## Results

### A substantial subset of CLL patients harbor more than one CDR3 peak

**Fig 1:**
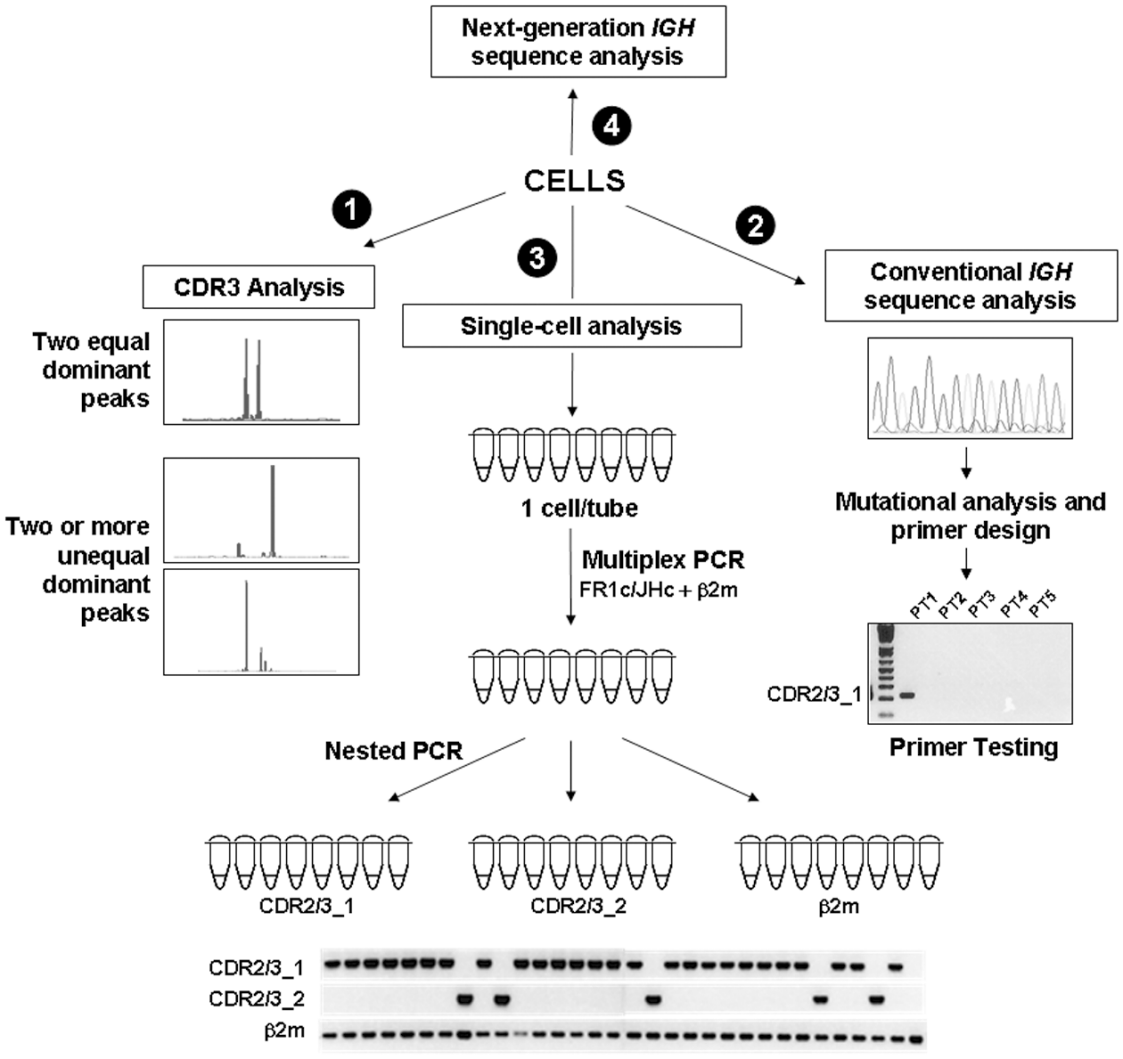
Flow chart for molecular analysis of CLL patients. (1) CDR3 analysis was initially performed to screen for patients exhibiting more than one dominant CDR3 peak. (2) Clonotypic sequences corresponding to dominant CDR3 peaks were characterized. Primers were designed based on unique sequences on the CDRs and were tested for specificity. (3) CLL cells were sorted in aliquots of 1, 10 or 100 cells and clonal analysis was performed by nested PCR using clone-specific primers. (4) Selected genomic DNA samples were subjected to next-generation *IGH* sequencing.

Typically, CLL is characterized by a monoclonal B-cell expansion yielding a single CDR3 peak profile in DNA fragment analysis, unlike the polyclonal profile usually seen in healthy donors. In a cohort of 198 CLL patients, CDR3 profiling identified 26 patients who exhibited 2–5 dominant CDR3 peaks, suggesting the presence of more than one B-cell clone and/or clones with biallelic *IGH* rearrangements. Table 2 shows the patients grouped by the number of *IGH* rearrangements identified. These 26 CLL were subjected to further molecular characterization as outlined in Fig 1.

**Table 2:** Frequencies of CLL patients grouped by number of dominant *IGH* rearrangements.

	No. of patients		
No. of dominant *IGH* rearrangements	U-CLL	M-CLL	Total
1	59	113	172
2	20	2	22
3–5	0	4	4
Total	79	119	198

### Multiple *IGH* rearrangements in each patient have mostly concordant mutation status

Clonotypic *IGH* sequences were characterized in all 198 patients. The most frequent rearranged *IGH* sequence was readily detected and disease was categorized as U-CLL or M-CLL, with the cutoff at 2% mutation. For the 26 patients with multiple sequences, all *IGHV* types were concordant in any given patient (i.e. all mutated or all unmutated) except for patient CLL-200 who had one mutated and one unmutated sequence. CLL-200 was designated as M-CLL based on the most abundant clone identified in subsequent single-cell studies. The *IGHV* gene usage and length of CDR3 were as expected of U-CLL and M-CLL (S1 Fig).

### *IGH* biallelic rearrangements and/or multiple clones in a subset of CLL patients

**Fig 2:**
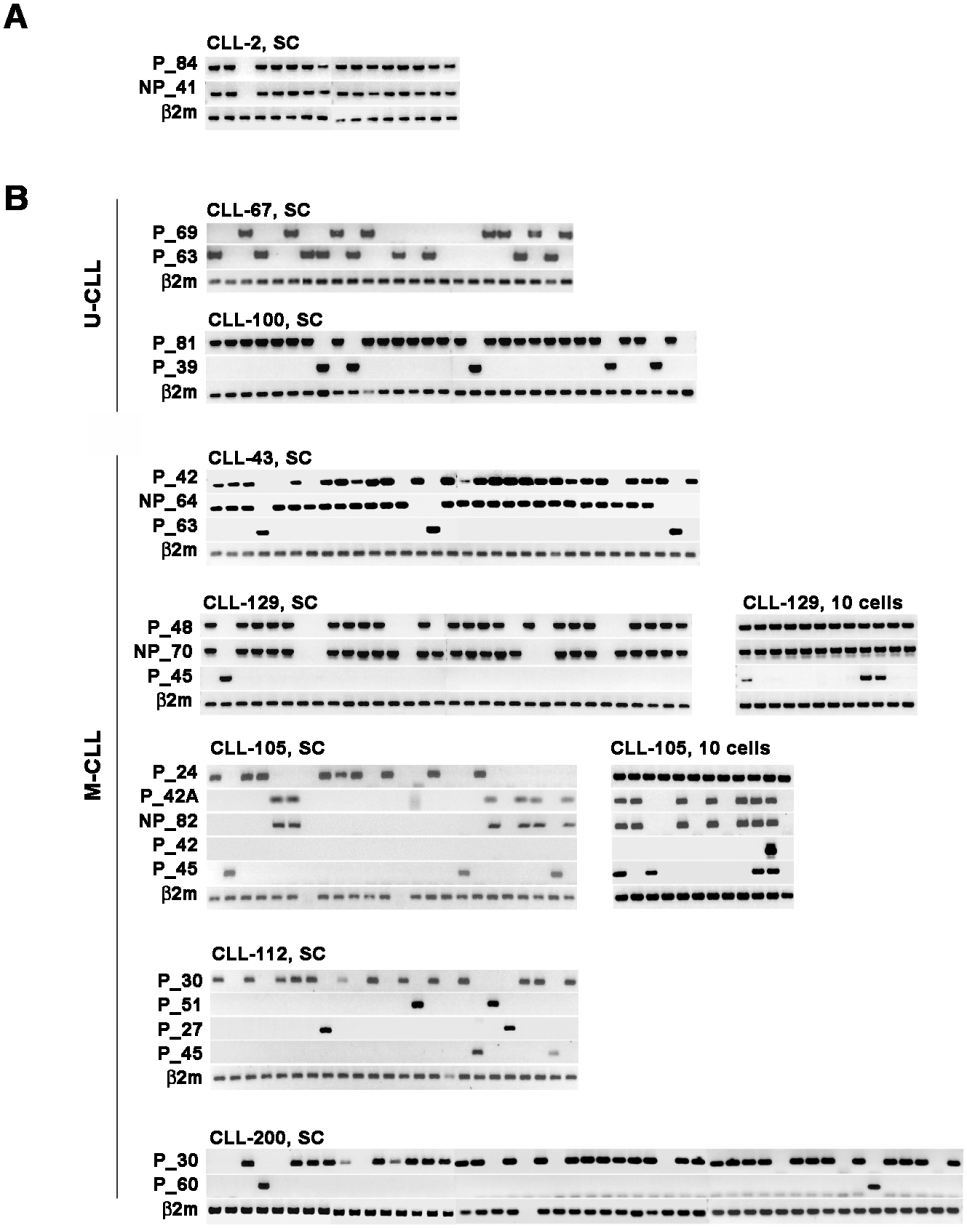
SCA identifies clonal origin of multiple rearranged heavy chain genes. (A) A representative result of biallelic rearrangements derived from single B-cell clone is shown in patient CLL-2. Each column represents nested PCR results of the same single cells. (B) Detection of clonal specific sequences in non-overlapping B-cell populations characterized biclonality or multiple clones (multiclonality). Biclonal or multiclonal B-cells comprised both monoallelic and biallelic clones. The number of single cells analyzed in each patient is larger than the number shown here. Analyses of 10-cell aliquots are shown in CLL-129 and CLL-105 to demonstrate and confirm the existence of clones that were infrequent. SC, single cell.

Of the 26 patients exhibiting more than one dominant CDR3 peak, 20 were in the U-CLL subgroup and 6 in the M-CLL subgroup; clinical features are shown in S1 Table. Representative results of SCA are shown in Fig 2.

Among U-CLL cases having two *IGH* rearrangements, each *IGH* rearrangement utilized a different *IGHV* gene (Table 3). For 18/20 patients, they comprised one P and one NP (i.e., out-of-frame or the presence of premature termination codons). SCA showed that these P/NP resided in the same cell (Fig 2A, patient CLL-2), which confirmed them as biallelic rearrangements in 7/7 patients analyzed (Table 3). Biclonality was confirmed in two other patients (CLL-67 and CLL-100, Fig 2B). For CLL-67, this was also supported by immunophenotypic analysis (data not shown).

**Table 3:** Characterization of rearranged *IGHV-IGHD-IGHJ* genes in U-CLL patients having two dominant *IGH* rearrangements.

	1^st^ *IGH*					2^nd^ *IGH*					
Patient ID	*IGH* ID^a^	*IGHV*	*IGHD*	*IGHJ*	*IGHV* mutation, %	*IGH* ID^a^	*IGHV*	*IGHD*	*IGHJ*	*IGHV* mutation, %	Clonal status determined by SCA
CLL-2	P_84	4–31	3–16	4	0	NP_41	3–53	5–5	1	0	biallelic
CLL-4	P_63	3–48	3–3	6	0	NP_71	3–30	3–3	6	0	biallelic
CLL-12	P_81	1–69	3–3	6	0	NP_47	3–30	2–21	4	0	biallelic
CLL-15	P_45	1–69	7–27	5	0	NP_58	1–45	3–9	4	0	biallelic
CLL-18	P_60	3–30	2–2	3	1.0	NP_64	3–13	2–21	4	2.0	ND
CLL-24	P_66	3–66	6–13	5	1.0	NP_95	3–9	3–10	1	1.4	ND
CLL-42	P_75	3–33	2–15	6	0	NP_46	3–21	6–13	5	0	ND
CLL-44	P_66	4–59	3–22	6	0	NP_44	1–45	3–10	4	0	ND
CLL-64	P_51	1–2	2–15	6	0.4	NP_46	3–74	6–13	4	0	ND
CLL-73	P_57	4–30	3–3	6	0	NP_33	1–18	4–23	5	1.7	biallelic
CLL-76	P_66	1–69	3–3	3	0	NP_60	3–9	3–22	2	0	ND
CLL-146	P_66	3–30	3–3	6	0	NP_46	4–59	6–13	6	0	ND
CLL-147	P_87	3–30	3–9	6	0	NP_46	4–39	3–22	4	0	ND
CLL-165	P_63	1–69	3–16	3	0	NP_75	1–46	3–16	3	0	biallelic
CLL-178	P_78	3–11	3–9	6	0	NP_53	3–23	2–2	5	0	ND
CLL-191	P_63	3–43	6–13	6	1.4	NP_69	NA	NA	NA	NA	ND
CLL-196	P_63	1–69	2–2	6	0	NP_83	2–70	2–2	4	0	biallelic
CLL-197	P_60	1–69	3–10	6	0	NP_71	4–40	2–15	6	0	ND
CLL-67	P_63	3–48	3–3	6	0	P_69	4–59	2–2	6	0	biclonal
CLL-100	P_81	1–69	3–16	5	0	P_39	3–74	3–10	4	0	biclonal

^a^ named by its productive (P) or non-productive (NP) status followed by the length of CDR3 in nucleotides.

NA, not fully characterized; ND, not done.

For the 6 M-CLL patients with more than one CDR3 peak (Table 4), one patient (CLL-40) had one biallelic P/NP clone. The other five patients had two or more partner B-cell clones (Fig 2B). The most abundant clone was designated as primary B-clone. CLL-43 and CLL-129 had a biallelic P/NP rearrangement in the primary clone and a second clone with a monoallelic P rearrangement. Patient CLL-105 had three distinct monoallelic clones and one clone with biallelic P/NP rearrangement. In this case, initial flow analysis had indicated three different CD5^+^ subsets: CD5^+^/kappa, CD5^+^/lambda and CD5^+^/polyclonal. Two clonal sequences, 105P_24 and 105NP_82, utilized the same *IGHV* gene segment but their different mutational profile and different *IGHD-IGHJ* gene usage argue against a shared origin (S2 Fig). A similar observation was also made for 105P_42 and 105P_45 (S2 Fig). For CLL-112, four monoallelic B-cell clones were identified, two of which were related (P_51 and P_27; S2 Fig). Patient CLL-200 had two distinct monoallelic clones.

**Table 4:** Characterization of rearranged *IGHV-IGHD-IGHJ* genes in M-CLL patients having two or more dominant *IGH* rearrangements and corresponding B-cell clones.

Patient ID	*IGH* ID^a^	*IGHV*	*IGHD*	*IGHJ*	*IGHV* mutation, %	No. of clones determined by SCA
CLL-40	P_60^b^	3–23	2–2	6	2.8	1
	NP_68^b^	4–59	3–22	4	2.1	
CLL-43	P_42^c^	3–7	3–3	4	8.2	2
	NP_64^c^	3–13	3–10	6	14.5	
	P_63	1–2	2–8	2	10.7	
CLL-105	P_24	3–7	3–16	3	3.1	4
	P_42A^d^	3–48	5–24	4	7.6	
	NP_82^d^	3–7	4–23	6	12.2	
	P_42	4–34	3–10	4	5.3	
	P_45	4–34	5–24	3	6.3	
CLL-112	P_30	4–4	2–21	4	7.3	4
	P_51^e^	1–08	2–2	6	6.2	
	P_27^e^	1–08	5–24	6	6.0	
	P_45	1–2	5–2	3	9.3	
CLL-129	P_48^f^	3–53	3–19	6	4.2	2
	NP_70^f^	1–2	3–16	4	5.2	
	P_45	3–30	3–3	4	5.9	
CLL-200	P_30	4–31	3–22	4	3.5^g^	2
	P_60	3–23	1–26	4	0	

^a^ named by its productive (P) or non-productive (NP) status followed by the length of CDR3 in nucleotides.

^b, c, d, f^ sequences coexisted in the same single cells (biallelic).

^e^ clonally related sequences

^g^ primary CLL clone.

Altogether, both biallelic rearrangements and/or multiclonality characterize a subset of patients with B-CLL. Each of the 37 B-cell clones analyzed from 26 patients had only one P *IGH* rearrangement per B-cell, meeting the restrictions of allelic exclusion. The incidence of biallelic rearrangements in the primary CLL clones was calculated to be 23% (18/79) in U-CLL, 2.5% (3/119) in M-CLL, or 10.6% (21/198) for the entire cohort. Biclonality occurred in 2.5% (2/79) of U-CLL while multiclonality (2–4 clones) was found in 4.2% (5/119) of M-CLL. Thus, biallelic rearrangements were more frequent in U-CLL than M-CLL (p<0.0001; Fisher’s exact test) but the incidence of bi/multiclonality was comparable (p = 0.7047; Fisher’s exact test). There was no statistical difference in mortality between patients with multiclonal *vs* monoclonal disease (S1 Text).

### Partner clones persist over time

Longitudinal studies were carried out in the seven CLL patients with more than one clone. SCA indicated that for 7/7 patients, multiple B-cell clones persisted over time (Tables 5 and 6). Partner clones are detected against a “background” of an abundant primary CLL clone with a B-cell count from 5x10^9^ cells/L to 312x10^9^ cells/L. For the CLL patients evaluated here, 9/11 (82%) partner clones had at one or more points in the disease a B-cell count >1x10^9^ cells/L (Tables 5 and 6). Five patients (CLL-43, CLL-67, CLL-100, CLL-105, CLL-112) harbored partner clones that at some point in time had a B-cell count >5x10^9^ cells/L (range = 5.8–28.7x10^9^ cells/L), fitting the working definition for a second CLL clones.

**Table 5:** Longitudinal analysis of *IGH* biclonality in U-CLL, as determined by SCA.

				Absolute count, x10^9^ cells/L (Clonal frequency, %)	
Patient ID	Years after diagnosis	Treatment status	TLC, x10^9^ cells/L	Clone 1	Clone2
CLL-67 ^a^	3	No	18.0	6.7 (37%)	4.7 (26%)
	6	No	34.0	13.7 (37%)	11.2 (33%)
	9	Yes	0.5	0.035 (7%)	0.003 (0.5%)
CLL-100 ^b^	5	Yes	312.0	190.0 (61%)	28.7 (9.2%)
	6	Yes	13.5	10.9 (81%)	2.1 (15.6%)

^a^ Clones 1, 67P_63; clone 2, 67P_69

^b^ Clones 1, 100P_81; clone 2, 100P_39

Underlined, second clone with absolute count >5x10^9^ cells/L.

**Table 6:** Longitudinal analysis of *IGH* biclonal and multiclonal diversity in M-CLL, as determined by SCA.

			Absolute count, x10^9^ cells/L (Clonal frequency)			
Patient ID	Years after diagnosis	TLC, x10^9^ cells/L	Clone 1	Clone2	Clone 3	Clone 4
CLL-43^a^	15	84	75.6 (90%)	1.3 (1.6%)	NA	NA
	16	96	76.8 (80%)	2.1 (2.2%)	NA	NA
	19	131	114.0 (87%)	5.8 (4.4%)	NA	NA
	20	120	118.0 (98%)	0.24 (0.2%)	NA	NA
	22	92	83.7 (91%)	4.1 (4.5%)	NA	NA
CLL-105^b^	2	12.9	7.0 (54%)	2.5 (19.5%)	0.8 (6.5%)	0.01 (0.1%)
	4	37	22.2 (60%)	6.1 (16.4%)	1.3 (3.6%)	0.3 (0.9%)
CLL-112^c^	0	26	20.1 (81%)	1.5 (5.7%)	1.4 (6%)	2.0 (8%)
	1	36.3	25 (69%)	6.9 (19%)	0.7 (2%)	1.1 (3%)
	3	51	33.1 (65%)	3.2 (6.2%)	2.1 (4.2%)	4.2 (8.3%)
	5	37	36.6 (99%)	0.15 (0.4%)	ND	ND
CLL-129^d^	1	10.4	9.8 (94%)	0.34 (3.3%)	NA	NA
	2	35	31.2 (89%)	0.56 (1.6%)	NA	NA
	2.5	45	43.2 (96%)	1.9 (4.2%)	NA	NA
	4	112	96.3 (86%)	2.6 (2.3%)	NA	NA
CLL-200^e^	4	11.1	8.0 (72%)	0.47 (4.2%)	NA	NA
	7	11	7.8 (71%)	0.24 (2.2%)	NA	NA

^a^ clone 1, 43P_42/NP_64; clone 2, 43P_63

^b^ clone 1, 105P_24; clone 2, 105P42A/NP_82; clone 3, 105P_45; clone 4, 105P_42

^c^ clone 1, 112P_30; clone 2, 112P_45; clone 3, 112P_27; clone 4, 112P_51

^d^ clone 1, 129P_47/NP_70; clone 2, 129P_45

^e^ clone 1, 200P_60; clone 2, 200P_30

NA, not applicable; ND, detectable in bulk sample but not frequent enough to be detected in single cells or 12x100-cell aliquots; underlined, second clone with absolute count >5x10^9^ cells/L.

For the two biclonal U-CLL patients (CLL-67 and CLL-100), partner clones were abundant (Table 5). Prior to treatment, CLL-67 had equivalent biclonal frequencies; treatment reduced the total lymphocyte count (TLC) from 34x10^9^ to 0.5x10^9^ cells/L, preferentially reducing the partner B-cell clone. In CLL-100, treatment reduced TLC from 312x10^9^ to 13.5x10^9^ cells/L, with both clones proportionately reduced.

None of the five patients in the M-CLL subgroup received treatment during the period of study (Table 6). For CLL-43, both major and minor clones persisted over a period of seven years, with samples taken between years 15 and 22 of the disease course. For CLL-105, CLL-129 and CLL-200, the ratios between the primary CLL clone and the partner clone(s) were consistent over time. In both CLL-105 and CLL-129, the absolute numbers for each clone continued to rise, with a steady increase in TLC. For CLL-112, the disease progressed during the third year, but the TLC remained relatively constant. By year 5, clonal dynamics in this patient led to preferential expansion of the primary clone.

### Confirmation of *IGH* multiclonality in CLL by NGS

**Fig 3:**
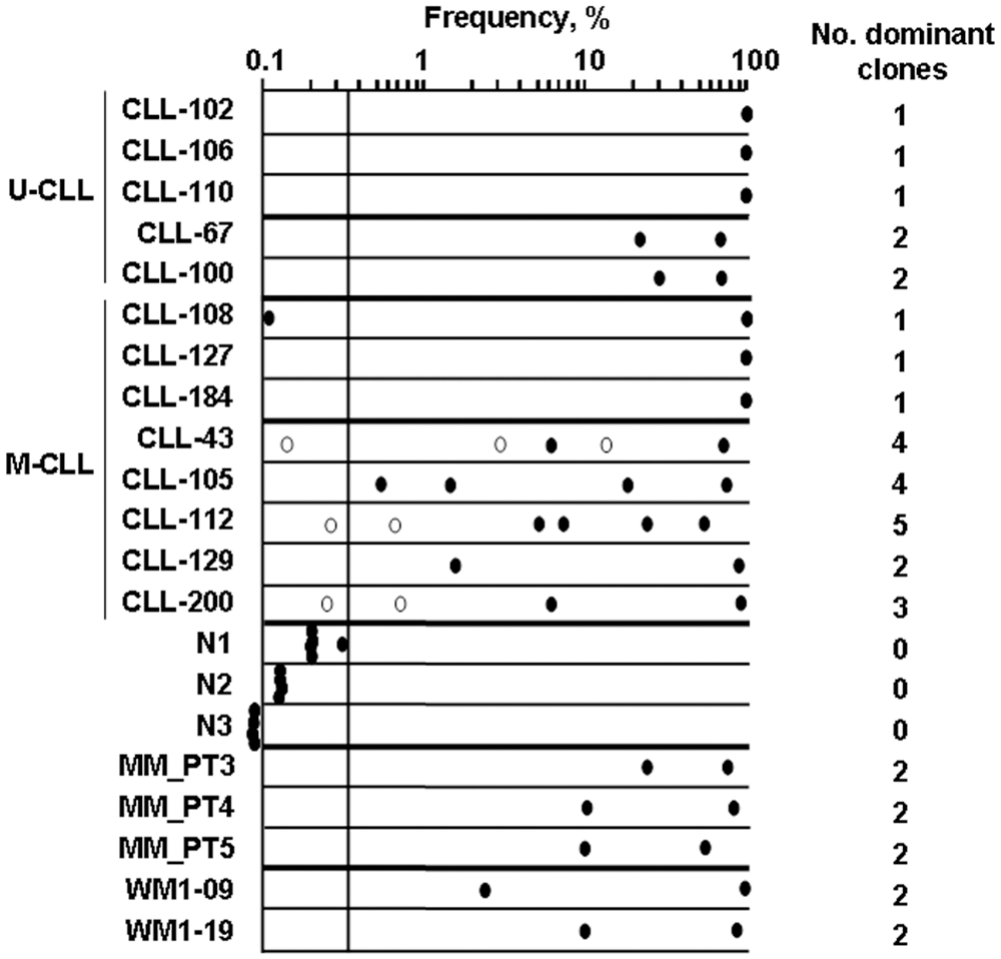
Multiclonality is frequently observed in M-CLL. *IGH* sequence frequencies were characterized by next-generation *IGH* sequencing and are plotted on log scale from 0.1–100%. Samples included a) seven CLL patients characterized as having more than one clone by SCA (U-CLL: CLL-67, CLL-100; M-CLL: CLL-43, CLL-105, CLL-112, CLL129, CLL200), b) six typical CLL with single B-cell clone (U-CLL: CLL-102, CLL-106, CLL-110; M-CLL: CLL108, CLL-127, CLL-184), c) three healthy donors (N1, N2, N3), and d) three MM and two WM patients in whom two B-cell clones were previously reported (MM_PT3, MM_PT4, MM_PT5, WM1-09 and WM1-19) [19, 20]. For sample N3, top frequencies were ≤0.035%, thus were placed outside of the *y*-axis for reference only, not to scale. An arbitrary cutoff line was drawn at the highest frequency found in HD. Dominant clones in CLL are defined as those with frequencies above the cutoff line. The number of dominant clones for each sample is shown on the right. Closed circle, clone identified by both ImmunoSEQ and SCA; open circle, clone identified only by ImmunoSEQ.

To validate our conventional clonal analyses, and to screen for any additional multiclonality, ImmunoSEQ analysis was performed on 13 CLL patients, including seven biclonal or multiclonal CLL and six “typical” CLL having a single dominant clone, three MM and two WM previously reported as biclonal,[19, 20] and three HD. ImmunoSEQ generated 2x10^5^-3x10^6^ reads/sample, giving 4x-70x coverage. The dataset includes sequence of CDR3 regions and part of *IGHV* sufficient for identifying the gene family. Clonal frequencies were calculated based on sequences of P rearrangements and are shown in Fig 3 and Table 7.

**Table 7:** Comparison of clonal frequencies estimated by SCA and ImmunoSEQ NGS.

			Clonal frequency, %	
Subgroup	Patient ID	Clone ID	SCA	ImmunoSEQ NGS^a^
U-CLL	CLL-67	67P_69	37	74^b^
		67P_63	33	23
	CLL-100	100P_81	61	70
		100P_39	9.2	29
M-CLL	CLL-43	43P_42/NP_64	87	74
		43P_63	4.4	6
	CLL-129	129P_48/NP_70	89	97
		129P_45	1.6	2.8
	CLL-200	200P_30	71	87
		200P_60	2.2	6.4
	CLL-105	105P_24	60	79
		105P_42A/NP_82	16.4	18.2
		105P_45	3.6	0.28
		105P_42	0.9	1.58
	CLL-112	112P_30	65	56
		112P_51	8.3	26
		112P_27	4.2	5.7
		112P_45	6.2	5.2

^a^ Frequencies calculated as percentage of total productive *IGH* rearrangements

^**b**^ 66% had complete sequence homology, 8% comprised intraclonal heterogeneity outside of the CDR3 region.

Overall, NGS found all B-cell clones identified by DNA fragment analysis and SCA, including the NP sequences. ImmunoSEQ also identified a small number of additional clones (open circles in Fig 3). For CLL-43, two more clones were identified. For CLL-112 and CLL-200, each patient had one additional clone. Clonal frequencies determined by NGS were generally consistent with those by SCA of the same sample (Table 7).

Although numbers are small, it is provocative that only M-CLL was accompanied by more than one partner clone for 4/5 patients analyzed (Fig 3). In contrast, even though both MM and WM also have mutated *IGHV*, each of the 5 patients analyzed had only one partner clone [19, 20]. Two biclonal U-CLL had only one partner clone, and control CLL classified as having only the primary CLL clone by conventional analysis, also had only one clone by ImmunoSEQ analysis.

## Discussion

Here we show that the presence of more than one rearranged *IGH* allele in CLL may be related to a P/NP rearrangement in the same cell, and/or to the presence of unrelated “partner clones” that coexist with the primary CLL clone. Although others have reported the presence of multiple clones in CLL, for the most part analysis was by immunophenotype or light chain restriction. This is the first report to demonstrate expansion of multiple B-cell clones in a subset of CLL patients, using single-cell analysis and next-generation *IGH* sequencing. While biallelic P/NP rearrangements were more frequent in U-CLL, the presence of more than one clone occurred with equal frequency in U-CLL and M-CLL. Interestingly, those cases with >2 clones were more frequent in M-CLL. In general, the secondary clones may represent coexisting MBL, although in some cases they were of sufficient size to constitute a second CLL clone.

Initial screening of 198 patients by CDR3 analysis identified 172 patients having a single rearranged *IGH* allele (monoallelic), and 26 patients who exhibited more than one rearranged *IGH* allele and/or more than one clone (biallelic, biclonal or multiclonal). Altogether, we analyzed 37 B-cell clones from 26 CLL patients: each B-cell clone carried only one productive *IGH* allele. In contrast to a previous study [3], we found no failures of allelic exclusion in this cohort of patients. We also identified a frequent subset of U-CLL patients whose clonal B-cells harbored a failed *IGH* rearrangement in addition to their productive *IGH* rearrangement. The proportion of U-CLL with biallelic rearrangements (23%) was comparable to the range in normal B-cell populations [26–28]. The biallelic rearrangement pattern was in contrast to that seen in M-CLL, MM or WM, most of which harbored one productive rearrangement and a presumptive germline allele. The frequency of P/NP rearrangements in patients with M-CLL was 10 fold lower (2.5%) than in U-CLL. Since memory B-cells are known to include those with biallelic P/NP [26], the parent B-cells that give rise to M-CLL appear to be negatively selected for biallelic *IGH*. This may reflect fundamental differences in the parent B-cells that give rise to M-CLL, MM and WM as compared to those giving rise to U-CLL or healthy memory B-cells.

Most previous reports of more than one rearrangement in CLL were not molecularly confirmed at the single-cell level but relied on phenotypic characterization, a less definitive clonal identifier. Here multiple clones were identified by two different methods and the findings were confirmed by evaluation of individual CLL cells. SCA indicated that all seven CLL having a productive plus a non-productive rearrangement were biallelic. For those CLL harboring more than one productive rearrangement, SCA confirmed that they represented two or more distinct B-cell clones.

The incidence of molecularly defined multiclonality in typical CLL shown here (7/198, 3.5%) was compatible with those reported by others for typical CLL [9]. However, this value is likely to be an underestimate because initial screening by CDR3 analysis in our study did not identify clones with equivalent length of CDR3. For multiclonal CLL reported here, the most abundant clone was designated as the primary CLL clone. Partner clones were consistently detectable for many years, at relatively constant ratios with the primary CLL clone. High count MBL may be little different from CLL. With this in mind, for several patients the absolute number of cells with the partner clonal rearrangement reached ≥5x10^9^ cells/L, the working definition for a second CLL clone in affected patients. The relatively frequent presence of partner clones suggests that evaluation of clonal heterogeneity and clonal evolution in CLL would benefit from inclusion of molecular analysis for *IGHV-IGHD-IGHJ* signatures to distinguish between intra-clonal and inter-clonal diversity. This would provide a means to identify minor clones with mutations, as detected by genome-wide analysis [17].

NGS analyzes the repertoire of B-cells in a large dataset that quantifies each clonal frequency. For comparison and as controls, we analyzed B-cells from CLL, MM, WM and HD. Monoallelic CLL identified by conventional means also scored as monoallelic by ImmunSEQ NGS, confirming its ability to discriminate biallelic and multiclonality in CLL. To distinguish the small increase in monoclonal B-cells found in HD from the considerably more abundant clonal B-cell expansions identified in CLL patients, an arbitrary cutoff was made above the highest frequency found in HD (Fig 3). Overall, both ImmunoSEQ and SCA yielded compatible frequencies (Table 7), except for CLL-67 in which SCA showed a lower frequency. This was likely due to clonotypic *IGHV* sequence heterogeneity found in one of the two clones (data not shown). However, ImmunoSEQ NGS could not replace SCA for verifying clonal identity.

In all CLL cases, clonal cells are present prior to the diagnosis of CLL, with multiclonality in low-count MBL [29–31]. In the majority of cases, only a single transformed clone reaches the threshold for massive clonal expansion. However, here we show that in five CLL patients who have at least one partner clone, the partner clone was sufficiently frequent at some points in time for designation as a second CLL. Thus, it may be clinically important to determine which clone harbors non-*IGHV* driver mutations identified by NGS. There is as yet no way to determine the clinical significance of partner clones. Nevertheless, their presence in a subset of CLL patients means that genome-wide sequencing analysis should address the contributions of inter-clonal diversity to genomic patterns.

## Supporting Information

S1 Fig *IGHV* distribution and length of CDR3 are different in U-CLL and M-CLL patients. *IGHV* gene family usage (A) and lengths of CDR3 (B) were compared between 79 U-CLL and 119 M-CLL. Biased *IGHV1* gene usage was seen in U-CLL subgroup (79/198 CCL) with half of *IGHV1* being *IGHV1-69*. The distribution of *IGHV* gene usage by M-CLL clones was comparable to the normal B-cell repertoire. Horizontal lines in (B) represent median values of CDR3 for each subgroup of patients. The U-CLL clones had longer CDR3 regions than did M-CLL clones (60.2±13.2, median = 63nt *vs* 45.7±11.9, median = 45nt; p<0.0001, Student’s *t*-test).
(TIF)

S2 Fig Clonal relationships were identified by comparing *IGHV* somatic mutation profiles. Three pairs of sequences were analysed: P24 *vs* NP82 (CLL-105, top panel), P42 *vs* P45 (CLL-105, middle panel) and P27 *vs* P51 (CLL-112, bottom panel). Clonotypic *IGHV* sequences were aligned to the closest germline sequence (A). Point mutations were shown and common base changes were boxed. Nucleotide sequence in the *IGHV-IGHD-IGHJ* junctions (B) and deduced amino acid sequence of CDR3 region (C) were also compared. Underlined nucleotides in *IGHD* gene segment indicated point mutations. Sequence homology in the *IGHV-IGHD-IGHJ* junctions was boxed. Dashes indicate identical nucleotides to germline sequence. Dots indicate gaps or nucleotides that are not taken into account for the alignments. *, stop codon; #, frameshift caused by N-addition that was not a multiple of 3.
(TIF)

S1 Table Clinical features of CLL patients with two or more dominant *IGH* rearrangements. (DOC)

S1 Text Clinical significance of biallelic or multiclonal disease. (DOC)
