# Supplementary figures and images for: Single-Cell Analysis and Next-Generation Immuno-Sequencing Show That Multiple Clones Persist in Patients with Chronic Lymphocytic Leukemia
Source: PLoS One. 2015 Sep 9;10(9):e0137232. doi: 10.1371/journal.pone.0137232 (PMC4564241; doi:10.1371/journal.pone.0137232)

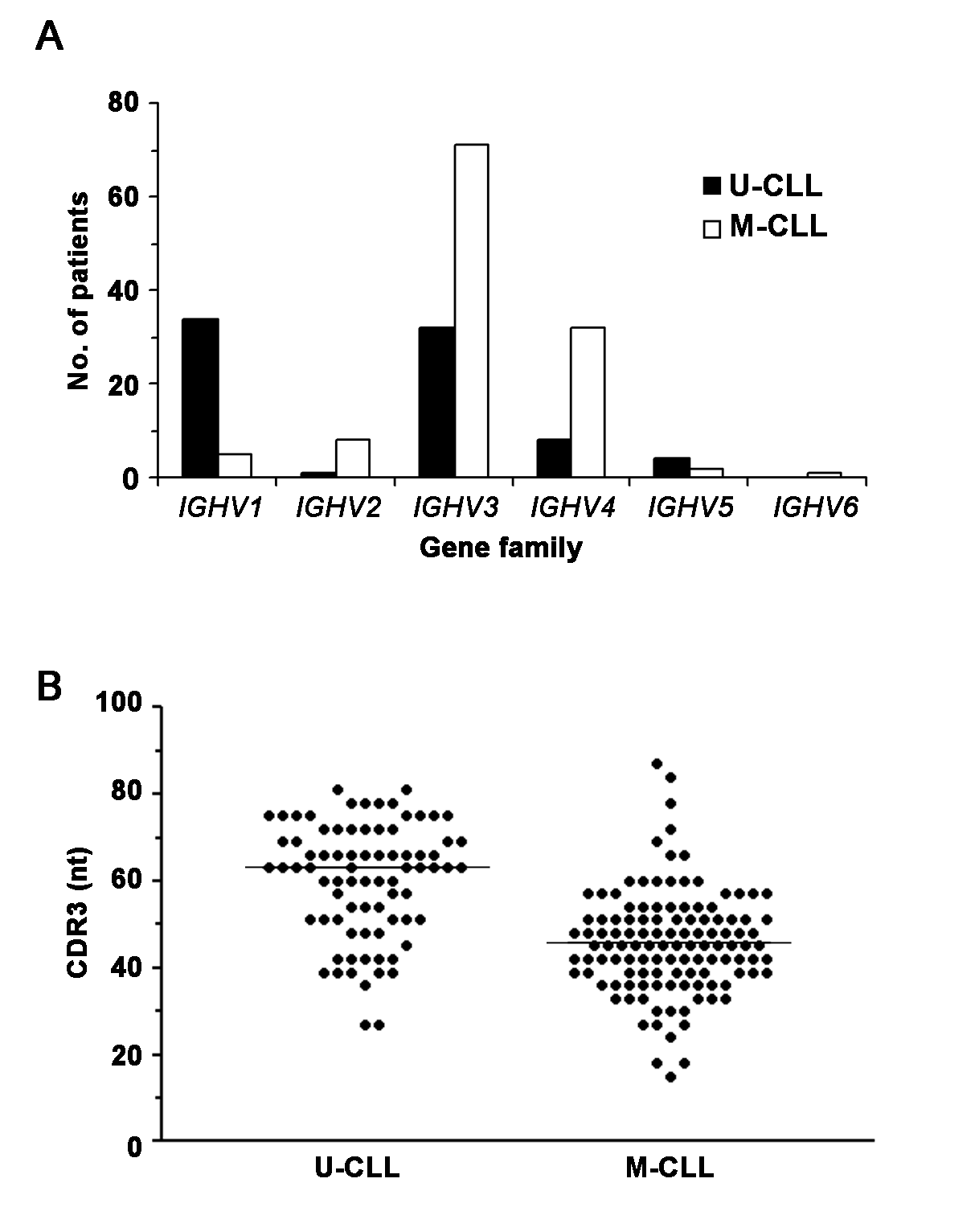

Supplement: S1 Fig — IGHV gene family usage (A) and lengths of CDR3 (B) were compared between 79 U-CLL and 119 M-CLL. Biased IGHV1 gene usage was seen in U-CLL subgroup (79/198 CCL) with half of IGHV1 being IGHV1-69. The distribution of IGHV gene usage by M-CLL clones was comparable to the normal B-cell repertoire. Horizontal lines in (B) represent median values of CDR3 for each subgroup of patients. The U-CLL clones had longer CDR3 regions than did M-CLL clones (60.2±13.2, median = 63nt vs 45.7±11.9, median = 45nt; p<0.0001, Student’s t-test). (TIF) [file pone.0137232.s001.tif]

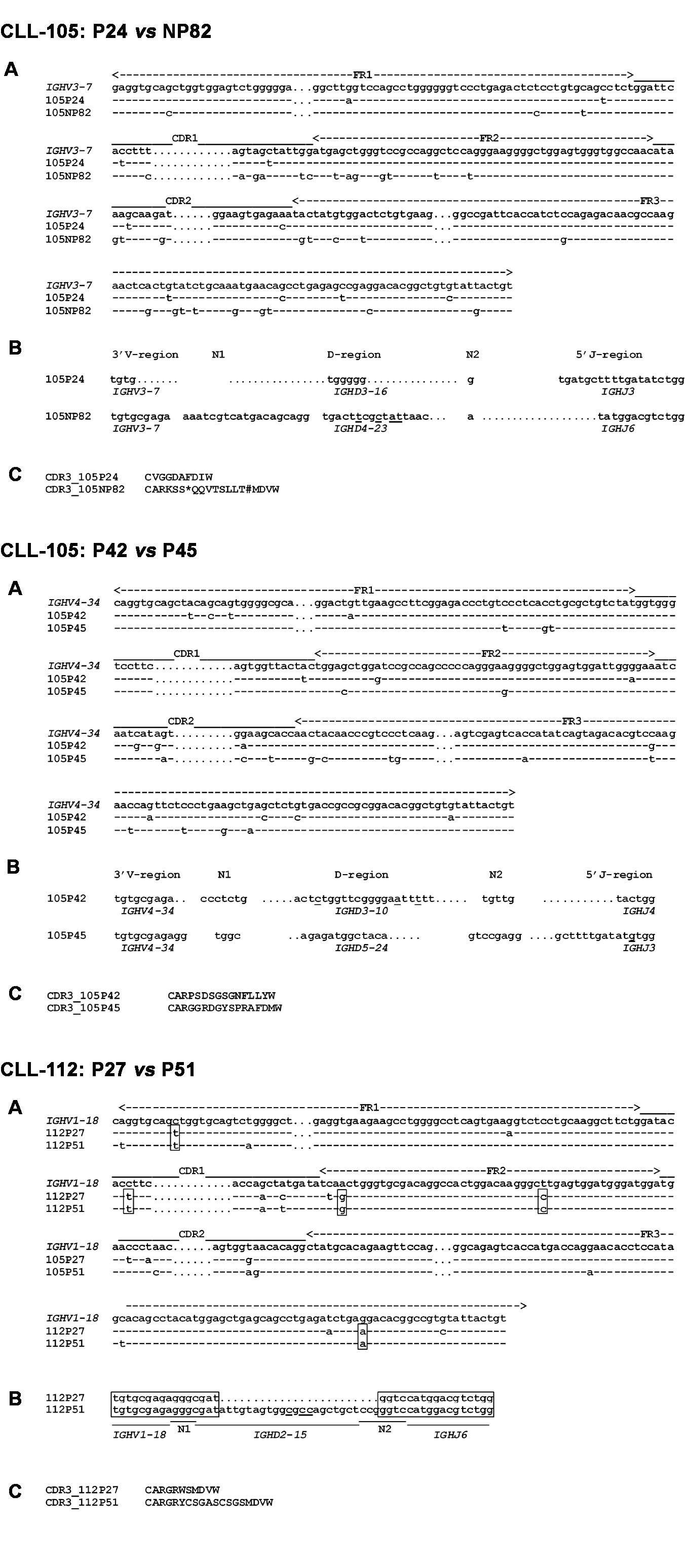

Supplement: S2 Fig — Three pairs of sequences were analysed: P24 vs NP82 (CLL-105, top panel), P42 vs P45 (CLL-105, middle panel) and P27 vs P51 (CLL-112, bottom panel). Clonotypic IGHV sequences were aligned to the closest germline sequence (A). Point mutations were shown and common base changes were boxed. Nucleotide sequence in the IGHV-IGHD-IGHJ junctions (B) and deduced amino acid sequence of CDR3 region (C) were also compared. Underlined nucleotides in IGHD gene segment indicated point mutations. Sequence homology in the IGHV-IGHD-IGHJ junctions was boxed. Dashes indicate identical nucleotides to germline sequence. Dots indicate gaps or nucleotides that are not taken into account for the alignments. *, stop codon; #, frameshift caused by N-addition that was not a multiple of 3. (TIF) [file pone.0137232.s002.tif]
