# Supplementary material for: Single-Cell Analysis and Next-Generation Immuno-Sequencing Show That Multiple Clones Persist in Patients with Chronic Lymphocytic Leukemia
Source: PLoS One. 2015 Sep 9;10(9):e0137232. doi: 10.1371/journal.pone.0137232 (PMC4564241; doi:10.1371/journal.pone.0137232)
Supplement: S1 Table — (DOC) [file pone.0137232.s003.doc]

**S1Table. Clinical features of CLL patients with two or more dominant *IGH*** rearrangements

| Subgroup | Patient ID | Age, yr^a^ | Sex | Rai Stage^a^ | CD-38, % | ZAP-70, % | Time between diagnosis and initial study | Treatment prior to initial study | Treatment following initial study | Status and time since initial study |
| --- | --- | --- | --- | --- | --- | --- | --- | --- | --- | --- |
| U-CLL | CLL-2 | 67 | M | 0 | 11 | 4 | 4.5 yr | Yes | Yes | Alive, 5.5 yr |
|  | CLL-4 | 55 | M | 0 | 1 | 6 | 2 yr | No | Yes | Dead, 7 yr |
|  | CLL-12 | 43 | F | 0 | 0 | 28 | 4 yr | Yes | Yes | Alive, 7 yr |
|  | CLL-15 | 79 | M | II | 35 | 2 | 6 yr | No | No | Dead, 2 yr |
|  | CLL-18 | 56 | F | 0 | ND | ND | 7.5 yr | Yes | Yes | Dead, 1 yr |
|  | CLL-24 | 81 | F | I | 0 | 100 | 8 yr | Yes | Yes | Alive, 7 yr |
|  | CLL-42 | 57 | M | I | ND | ND | 10 yr | Yes | Yes | Dead, 1.5 yr |
|  | CLL-44 | 59 | M | 0 | 95 | ND | 7.5 yr | Yes | Yes | Alive, 10 yr |
|  | CLL-64 | 66 | F | 0 | 0 | ND | 11.5 yr | Yes | Yes | Dead, 2 mo |
|  | CLL-73 | 84 | F | 0 | 0 | 53 | 1 mo | No | Yes | Alive, 5 yr |
|  | CLL-76 | 65 | M | 0 | 57 | 7 | 6 mo | No | Yes | Alive, 8 yr |
|  | CLL-146 | 69 | M | IV | 21 | 44 | 1 wk | No | Yes | Dead, 1 yr |
|  | CLL-147 | 49 | M | II | 19 | 0 | 2 mo | No | Yes | Dead, 2.5 yr |
|  | CLL-165 | 75 | M | I | 0 | 0 | 6 mo | No | Yes | Alive, 3.5 yr |
|  | CLL-178 | 76 | M | 0 | 0 | 44 | 3 yr | No | Yes | Dead, 5.5 yr |
|  | CLL-191 | 78 | M | III | 0 | ND | 9 yr | Yes | Yes | Dead, 2 yr |
|  | CLL-196 | 72 | M | 0 | 0 | 11 | 2 yr | No | Yes | Alive, 6 yr |
|  | CLL-197 | 48 | M | 0 | 30 | 28 | 6 yr | Yes | Yes | Dead, 6 yr |
|  | CLL-67^b^ | 60 | M | I | 5 | 82 | 3 yr | No | Yes | Alive, 8 yr |
|  | CLL-100^b^ | 49 | F | I | 0 | 9 | 5 yr | Yes | Yes | Alive, 6 yr |
| M-CLL | CLL-40 | 73 | F | II | 0 | 35 | 10 yr | Yes | No | Dead, 3.5 yr |
|  | CLL-43^b^ | 78 | M | 0 | 0 | 1 | 19.5 yr | No | No | Alive, 6 yr |
|  | CLL-129^b^ | 60 | F | 0 | 0 | 0 | 3.5 yr | No | No | Alive, 3.5 yr |
|  | CLL-200^b^ | 65 | M | 0 | 30 | 1 | 3.5 yr | No | No | Alive, 6.5 yr |
|  | CLL-105^b^ | 79 | F | 0 | 0 | 0 | 2 yr | No | Yes | Alive, 6 yr |
|  | CLL-112^b^ | 58 | M | I | 0 | 9 | 1 yr | No | No | Alive, 6 yr |

^a^ Age and Rai stage were those at the time of the first sample collection

^b^ Patients with two or more clones

ND, not done
