# Supplementary material for: Single-Cell Analysis and Next-Generation Immuno-Sequencing Show That Multiple Clones Persist in Patients with Chronic Lymphocytic Leukemia
Source: PLoS One. 2015 Sep 9;10(9):e0137232. doi: 10.1371/journal.pone.0137232 (PMC4564241; doi:10.1371/journal.pone.0137232)
Supplement: S1 Text — (DOC) [file pone.0137232.s004.doc]

## S1 Text. Clinical significance of biallelic or oligoclonal disease

Biallelic rearrangements were highly associated with U-CLL whereas CLL cases exhibiting more than 2 clones was associated with M-CLL (p<0.0001). Compared to patients with multiclonal disease, patients having a biallelic condition were more likely to need treatment (p=<0.001). Odds Ratio estimates showed that patients with biallelic disease were 40 times more likely to be U-CLL (95% confidence interval (CI) 5.275-309.243) and 12 times more likely to require treatment (95% CI 1.558-91.093) than patients with non-biallelic disease. Logistic regression models showed a 2.9-fold increase in risk of mortality for biallelic patients compared to patients who were not biallelic (95% CI 1.133-7.422). However, when biallelic patients were compared to monoallelic U-CLL patients, there was no difference in time to treatment or survival, indicating that the poor prognosis of these patients was related to the fact that most were unmutated. It is the mutational status and not the rearrangement that associates with aggressive disease.
